# Techniques for Injection of the Scaphotrapezium-Trapezoid Joint Without Image Guidance

**DOI:** 10.1016/j.jhsg.2021.05.003

**Published:** 2021-06-08

**Authors:** Casey Imbergamo, Amr Tawfik, Brian Bueno, Emily Van Kouwenberg, Ajul Shah, Brian M. Katt

**Affiliations:** ∗ Rutgers Robert Wood Johnson Medical School, New Brunswick, NJ; † Center for Hand and Upper Extremity Surgery, Shrewsbury, NJ

**Keywords:** Cadaveric, Joint injection, Palpation, Scaphotrapezium-trapezoid, STT

## Abstract

**Purpose:**

Scaphotrapezium-trapezoid (STT) joint arthritis is one of the most common forms of wrist arthritis. Conservative management often involves corticosteroid injection. Despite this, there is a scarcity of literature on palpation-guided injection techniques for the STT joint. We aimed to determine a standardized palpation-guided injection method that is easily reproducible and poses minimal risk to local anatomic structures.

**Methods:**

Six fresh-frozen cadaveric upper extremity specimens were tested. Access to the STT joint was attempted with dorsal, volar, and radial approaches. Fluoroscopy was used to confirm accurate placement within the joint. Needle placement was documented in relation to the surrounding soft tissue and bony landmarks were measured with a ruler, and the angle of the needle entry was recorded using a goniometer. The cadavers were carefully dissected to identify the surrounding neurovascular structures at risk of injury.

**Results:**

To access the STT joint with the dorsal approach, the needle was angled at 90º and inserted one-third of the distance from the prominence of the base of the second metacarpal to Lister tubercle. No neurovascular structures were found in the immediate vicinity of the needle. For the volar approach, the needle was angled at 65º and inserted at the distal wrist crease, 1 cm ulnar to the radial border of the wrist, in line with the second metacarpal. The volar branch of the radial artery was at risk with this approach. For the radial approach, the needle was angled at 60º and inserted immediately dorsal to the extensor pollicis brevis tendon, midway between the radial styloid and the prominence of the thumb metacarpal base. The dorsal branch of the radial artery was at risk with this approach.

**Conclusions:**

In a clinical setting where fluoroscopy or ultrasound is not readily available, the dorsal approach may allow for safe and accurate placement of the injectate into the STT joint.

**Type of study/level of evidence:**

Therapeutic IV.

Scaphotrapezium-trapezoid (STT) arthritis is a common form of arthritis in the wrist, with a reported incidence of 15% to 59% on radiographic evaluation.^1^^,^^2^ Scaphotrapezium-trapezoid arthritis most frequently affects middle-aged and elderly patients. It commonly causes severe pain and impairment of daily function.^3^^,^^4^ Patients most often present with radial-sided wrist pain that worsens with movement, decreased range of motion, reduced grip strength, and localized swelling.^3^^,^^4^ Physical examination findings suggestive of STT arthritis are also found in those patients with trapeziometacarpal arthritis, thus making the diagnosis of STT arthritis difficult using physical examination findings alone. One method that has shown promise diagnostically is the injection of lidocaine into the STT joint. It has been found that in cases of isolated STT arthritis, pinch strength improves immediately following injection.^5^ The role of injection into the STT joint in patients with STT arthritis goes beyond diagnosis, as the injection of corticosteroid is a common method of nonsurgical treatment for these patients.^6^ Other treatments include activity modification, orthosis fabrication with long and/or short opponens orthoses, and the use of nonsteroidal anti-inflammatory drugs.^6^ If initial treatment with these methods fails, a more invasive surgical approach may be warranted. Such procedures include arthrodesis, distal scaphoid excision, implant arthroplasty, and trapeziectomy.^3^

As intra-articular injections play an important role in the diagnosis and treatment of STT arthritis, we sought to explore methods of STT joint injection. Previous studies have outlined the use of ultrasound- or fluoroscopic-guided STT joint injections to ensure accurate needle placement into the joint.^7^ However, there is a scarcity of existing literature pertaining to palpation-guided injections of the STT joint, with 1 cadaveric study reporting the accuracy of a dorsal approach to be 80%.^8^ The purpose of this study was to explore and describe techniques for palpation-guided STT joint injections using dorsal, volar, and radial approaches in cadaveric specimens. We aimed to determine a standardized palpation-guided injection technique that is easily reproducible and poses minimal risk to local anatomic structures. The null hypothesis of this study was that there would be no difference in success rates when accessing the joint from a dorsal, volar, or radial approach.

## Materials and Methods

This study was completed in the Rutgers Robert Wood Johnson Hospital Procedural Skills Laboratory. Cadaveric specimens were obtained from the Musculoskeletal Transplant Foundation, Edison. Six fresh-frozen cadaveric upper extremity specimens were thawed at room temperature immediately prior to use. The specimens were inspected for signs of trauma or postsurgical changes affecting the wrist and were found to be free of such changes on clinical and radiographic evaluation. The mean cadaver age was 78 years (range, 65–91 years), with 4 right-sided specimens (2 male and 2 female) and 2 left-sided specimens (female).

A 21-gauge needle was used to access the STT joint from dorsal, volar, and radial approaches. Each approach was attempted on all 6 cadaveric specimens, for a total of 18 attempts. The order of approaches used was varied with each cadaver to minimize bias. Following needle placement, a Philips BV Pulsera mobile fluoroscopic imaging system (Philips Healthcare) was used to confirm the accuracy within the joint. Needle placement was documented in relation to the surrounding soft tissue and bony landmarks and distances were measured. Additionally, the angle of the needle entry was recorded using a goniometer against the horizontal plane. With the needle remaining in place, the wrist was then carefully dissected to identify the surrounding vasculature and nerves that were at risk in the immediate vicinity of the needle. Needle placement, measurement of anatomic landmarks, and dissection were performed by a fellowship-trained hand surgeon (B.K.).

After documenting the palpation-guided approaches in the first cadaveric specimen, the STT joints of all 6 wrists were successfully accessed using the same anatomic landmark-based protocols. These needle placements were performed by medical students (C.I., A.T., and B.B.) who had minimal experience with small joint injection techniques. Following needle placement, fluoroscopic imaging was again used to confirm the accuracy within the joint. The number of attempts to successfully insert the needle into the STT joint was recorded for each of the 3 approaches, as defined by the number of times the needle had to be reinserted or repositioned. Three additional specimens were again dissected to identify the at-risk anatomic structures with each approach. Dissection was done after needle insertion was attempted from each of the 3 approaches to avoid any distortion of the normal anatomy prior to needle placement. After completing all attempts for approaches to the STT joint in each of the cadavers, 2 nondissected specimens were injected with contrast dye to determine the presence of a potential communication between the STT and midcarpal joints.

## Results

### Dorsal approach

**Figure 1:**
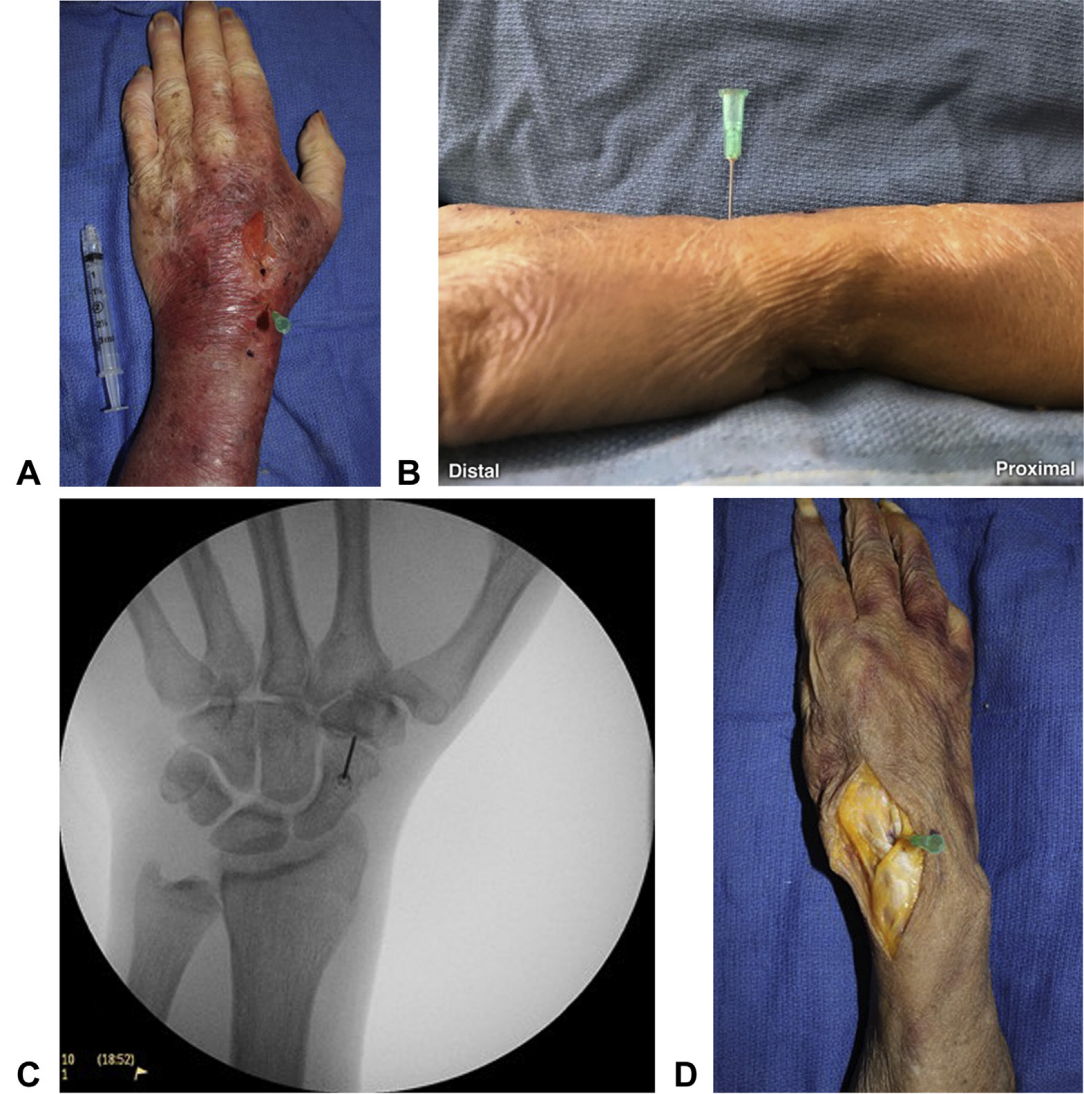
**A** Dorsal approach landmarks: the base of the second metacarpal and Lister tubercle. **B** Needle insertion angle with dorsal approach. **C** Fluoroscopic confirmation of accurate needle placement in the STT joint with the dorsal approach. **D** Dissection of the specimen accessed with dorsal approach: needle placement radial to the extensor carpi radialis brevis tendon and ulnar to the extensor carpi radialis longus tendon.

In the dorsal approach, the cadaveric hand was held in a pronated and flat position. The prominence of the base of the second metacarpal and Lister tubercle were identified by palpation and were subsequently marked (Fig. 1A). Lister tubercle was chosen as a landmark as it separates the second and third extensor compartments, allowing for the identification of the extensor pollicis longus, extensor carpi radialis brevis, and extensor carpi radialis longus tendons. The midportion of the flare of the base of the second metacarpal was chosen because of its ease of identification and longitudinal relationship with Lister tubercle. The needle was inserted at a 90º angle from the horizontal plane at a point one-third of the distance from the prominence of the base of the second metacarpal to Lister tubercle (Fig. 1B), along the line between these 2 structures. The average distance between these landmarks was 4 cm, with the insertion point being 1.4 cm proximal to the base of the second metacarpal. The needle was advanced volarly until a capsular puncture was distinctly felt. After the capsular puncture, the location of the needle tip was confirmed by fluoroscopy (Fig. 1C). The dorsal approach was attempted on all 6 cadavers, with the number of discrete attempts (defined as having to reinsert or reposition the needle) recorded for each specimen. The dorsal approach required a mean of 1.50 attempts (range, 1–4 attempts) to successfully insert the needle into the joint space (Table). The dissection revealed that the needle tip was inserted radial to the extensor carpi radialis brevis tendon and ulnar to the extensor carpi radialis longus in all specimens (Fig. 1D). With this approach, the radial artery was not at risk of puncture in any of the cadavers.

**Table:** Attempts Required to Successfully Access the STT Joint Using Each Approach

Approach	Average Number of Attempts	Specimen 1	Specimen 2	Specimen 3	Specimen 4	Specimen 5	Specimen 6
Dorsal	1.50	1	1	1	1	1	4
Volar	2.33	2	3	2	2	2	3
Radial	1.66	2	1	2	1	3	1

### Volar approach

**Figure 2:**
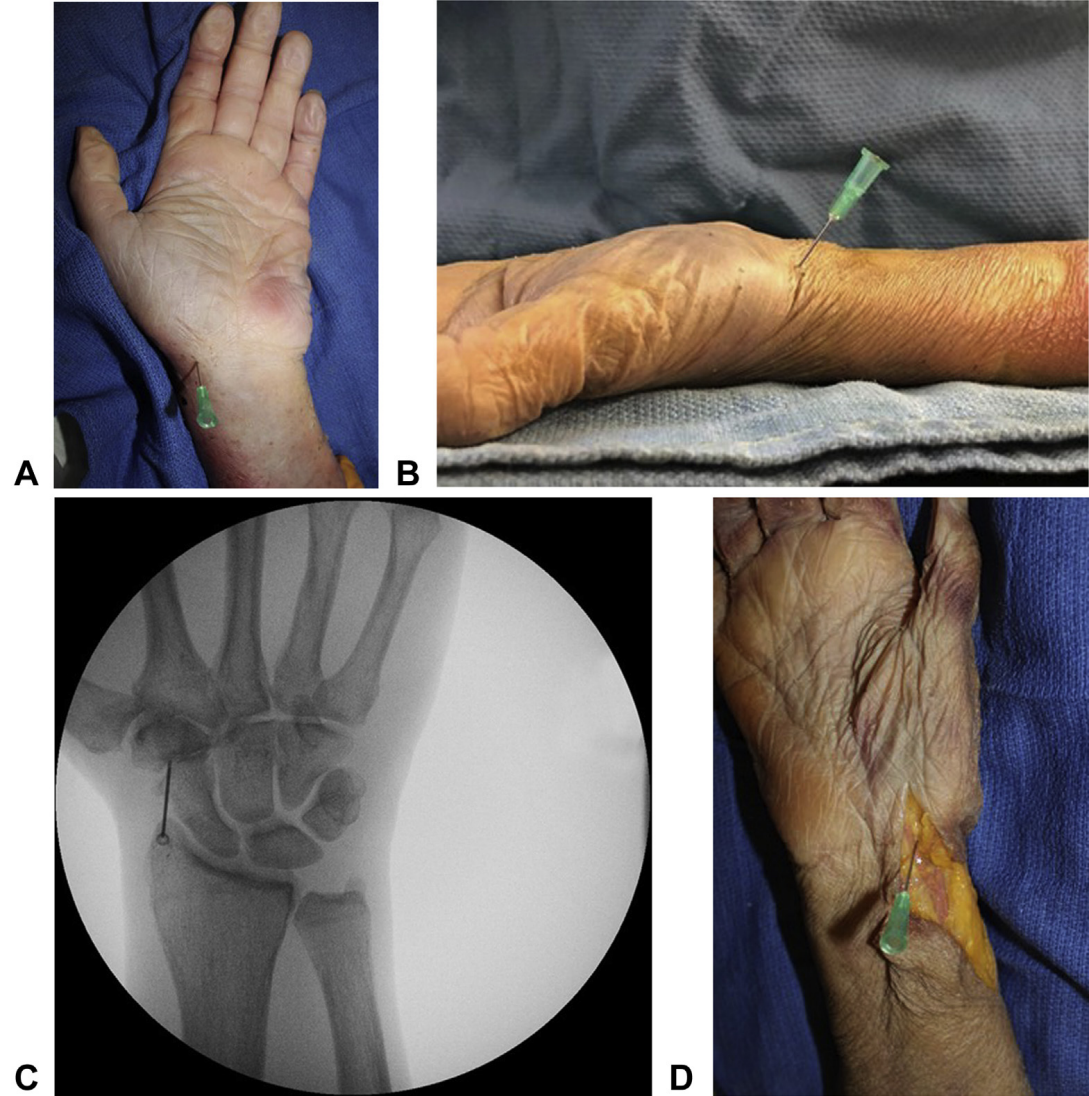
**A** Volar approach needle insertion point: at the distal wrist crease, 1 cm ulnar to the radial border of the wrist, in line with the second metacarpal. **B** Needle insertion angle with volar approach. **C** Fluoroscopic confirmation of accurate needle placement in the STT joint with the volar approach. **D** Dissection of the specimen accessed with volar approach: needle placement radial to the volar branch of the radial artery.

In the volar approach, the hand was placed in a fully supinated position, resting flat on the table. The starting point for the needle was at the distal wrist crease, 1 cm ulnar to the radial border of the wrist and in line with the second metacarpal (Fig. 2A). The needle was angled at 65º from the horizontal plane, pointing distally (Fig. 2B). The needle was advanced until a capsular puncture was distinctly felt. After the capsular puncture, the location of the needle tip was confirmed by fluoroscopy (Fig. 2C). The volar approach was attempted on all 6 cadavers, with the number of discrete attempts (defined as having to reinsert and reposition the needle) recorded for each specimen. The volar approach required a mean of 2.33 attempts (range, 2–3 attempts) to successfully insert the needle into the joint space (Table). The dissection revealed that the needle placement was immediately radial to the volar branch of the radial artery, which is the anatomic structure primarily at risk with this approach, in all specimens (Fig. 2D).

### Radial approach

**Figure 3:**
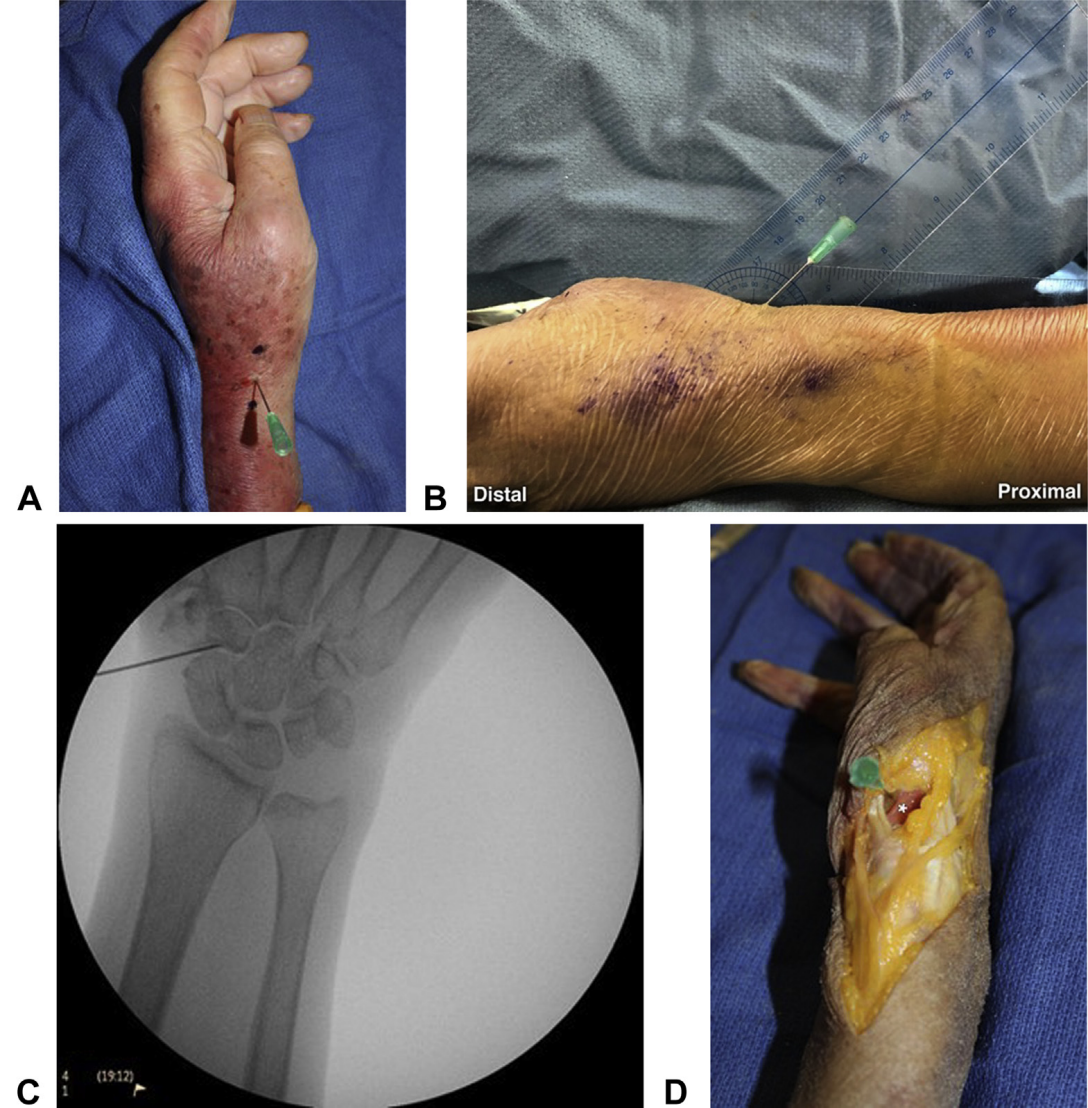
**A** Radial approach needle insertion point: immediately dorsal to extensor pollicis brevis tendon, midway between the radial styloid and the prominence of the thumb metacarpal base. **B** Needle insertion angle with radial approach. **C** Fluoroscopic confirmation of accurate needle placement in the STT joint with the radial approach. **D** Dissection of the specimen accessed with radial approach: needle placement radial to the dorsal branch of the radial artery.

In the radial approach, the hand was rested on the table along its ulnar side with the wrist in a neutral position. The starting point for the needle was immediately dorsal to the extensor pollicis brevis tendon, midway between the radial styloid and the prominence of the thumb metacarpal base (Fig. 3A). The average distance between these landmarks as measured was 3 cm, with the insertion point being 1.5-cm equidistant from both landmarks. The needle was angled at 60º from the horizontal plane, pointing distally (Fig. 3B). The needle was advanced until a capsular puncture was distinctly felt. After the capsular puncture, the location of the needle tip was confirmed by fluoroscopy (Fig. 3C). The radial approach was attempted on all 6 cadavers, with the number of discrete attempts (defined as having to reinsert and reposition the needle) recorded for each specimen. The radial approach required a mean of 1.66 attempts (range, 1–3 attempts; Table). The dissection revealed that the needle placement was immediately dorsal to the dorsal branch of the radial artery, which is the anatomic structure primarily at risk with this approach, in all specimens (Fig. 3D).

### Radiopaque dye injection

**Figure 4:**
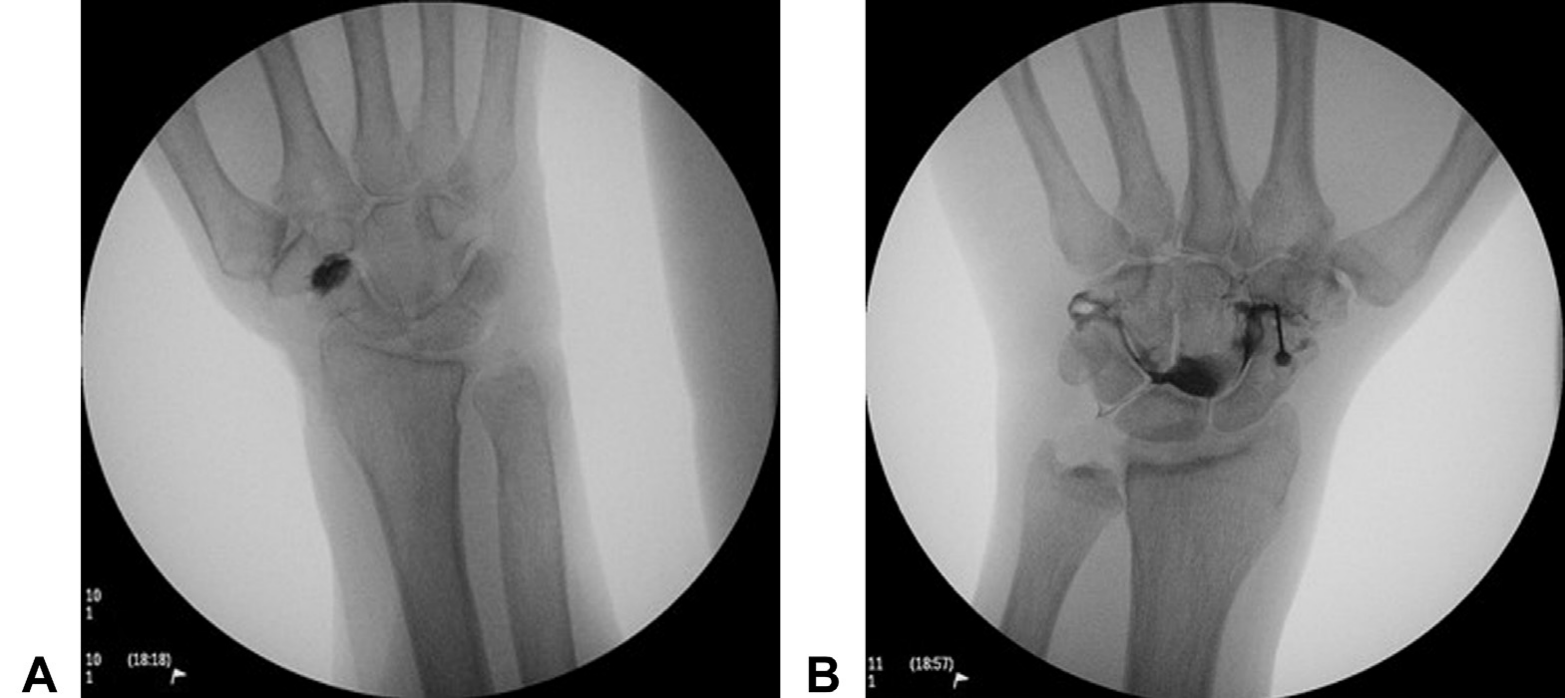
**A** Radiopaque dye was injected into the STT joint. **B** Radiopaque dye was injected into the STT joint, dispersing into the midcarpal joints.

After attempting each of the 3 approaches to the STT joint in all 6 cadavers, iohexol was injected into the joint spaces of 2 specimens using the dorsal approach. The dorsal approach was used, as it was found to have the highest success rate in accessing the joint during our testing. After intra-articular needle placement was confirmed by fluoroscopy, a 1-mL syringe was used to inject the contrast dye until resistance was met or until the injection of full 1 mL was completed. The placement of the dye was then evaluated using fluoroscopy. In 1 specimen, 0.4-mL dye was injected until resistance was felt. Fluoroscopy then confirmed that the dye remained localized to the STT joint and tissues immediately surrounding it. (Fig. 4A). In the second specimen, full 1-mL dye was injected without resistance. Fluoroscopy revealed that the contrast dye had dispersed from the STT joint into the midcarpal joints (Fig. 4B).

## Discussion

Since corticosteroid injections are a potentially beneficial method of treatment for patients with STT arthritis, proper injection into the intra-articular space must be reliably achieved.^6^ Smith et al^8^ compared palpation-guided and ultrasound-guided techniques for injection of the STT joint space. The authors found that the injectate was successfully placed into the joint with 100% accuracy using the ultrasound-guided technique, whereas 80% of attempts were successful with the palpation-guided technique. Though ultrasound was proven to be a more effective method of injection, such imaging is not always readily available. Therefore, a reproducible and explicitly described palpation-guided technique that provides a successful intra-articular injection is needed. We believe that the dorsal approach described in this study will allow providers to achieve an accuracy that is equal to or greater than the palpation-only rates described previously in the literature.^8^ Our use of specific anatomic landmarks such as Lister tubercle and the base of the second metacarpal allow for a more definitive approach with regard to the wrist anatomy and entry point of the needle. In comparison, Smith et al^8^ described palpating the extensor pollicis longus tendon and base of the anatomic snuff box to guide injection, without more specific landmarks or distances to the point of entry.

We propose our dorsal approach as a reliable technique for injections of the STT joint without requiring image guidance. The dorsal approach affords the most safety of the 3 approaches, as there is minimal risk of inadvertent injury to vasculature or nerves in the immediate field. The previously described palpation-guided techniques entered the STT joint radial to the extensor pollicis longus tendon at the base of the anatomic snuffbox, leaving the dorsal branch of the radial artery at risk of puncture.^8^ In comparison, our technique enters the STT joint between the extensor carpi radialis brevis and extensor carpi radialis longus tendons, clear from any major neurovascular structures. The volar and radial approaches explored in this study share similar safety concerns. Dissection of the wrist following needle insertion with the volar and radial approaches showed close proximity of the needle paths to the volar and dorsal branches of the radial artery, respectively. Though injury to these vessels was avoided in this study, a live patient would have circulating blood, leading to an increased vessel circumference and increased risk of injury. Additionally, successful insertion of the needle into the joint capsule using the volar and radial approaches required repositioning of the needle tip distally and proximally, furthering the potential risk to nearby structures. The volar and radial approaches required more attempts on average to successfully enter the STT joint compared with the dorsal approach. Regarding the volar approach, bone spurs on the volar aspect of the STT joint may have made entry with this technique more difficult. The palpation-guided technique described here provided the proper insertion point and entry angle; however, the needle had to be repositioned with fine movements in order to access the joint. Regarding the dorsal approach, with the exception of 1 specimen with a particularly arthritic STT joint, successful insertion into the STT joint with the dorsal approach was achieved with a single attempt without repositioning. In comparison, the radial approach had a 50% success rate of accessing the joint with a single attempt without repositioning, versus 83% for the dorsal approach. Therefore, in a clinical setting in which fluoroscopy or ultrasound is not available, the dorsal approach described in this study allows for safe and accurate placement of injectate into the STT joint.

Finally, the injection of the dye into the STT joint using the dorsal approach successfully resulted in the retention of the dye within the joint space, as confirmed by fluoroscopy. The first attempt resulted in the injection of 0.4-mL dye, at which point resistance was felt and the injection was ceased. Fluoroscopy revealed that the dye was retained within the STT joint space and showed no signs of communication to other joints within the wrist. With the second attempt of injection, no resistance was encountered, and the entire 1-mL dye was injected. Fluoroscopy revealed that the dye was not confined to the STT joint space and dispersed throughout the adjacent joints. It has been noted in the literature that anatomic communications exist between the joints of the wrist, such as the STT and midcarpal joints.^8^^,^^9^ It is possible that our findings can be attributed to anatomic variation of the STT joint among the specimens; however, this is a concept that warrants further investigation. In a clinical setting, if a patient with STT arthritis does have a communicating joint and a steroid injection is inadvertently placed into a nearby joint, it may still disperse into the STT joint, providing the desired intra-articular effect.

Our study has several limitations that should be mentioned. Primarily, needle placement in cadaveric specimens does not fully simulate the placement in a patient. Although the tissue quality of a fresh-frozen cadaver is the most optimal tissue available for anatomic studies, there are still differences that must be considered when extrapolating the results to a clinical setting. Patients have active muscle tone, and their tendons may be under tension during the injection. This could potentially allow clinicians to more easily identify the extensor tendons on the dorsal surface of the hand when determining needle placement. In a cadaver, such factors are not encountered. It is also important to note that in a clinical setting, joint injections are painful for patients, and any repositioning of the needle would cause discomfort. This further supports the use of the dorsal approach to the STT joint, as it required the least amount of needle repositioning and may therefore minimize discomfort for patients. Additionally, the variation in human anatomy may result in minor inconsistencies in our palpation-based approach from patient to patient. However, these variations in anatomy are at least partially addressed by our use of ratios and bony landmarks rather than specific metric measurements.

In conclusion, the findings of this research may help providers use anatomic landmarks to confidently perform injections into the STT joint space, particularly in settings where ultrasound or fluoroscopic guidance may not be readily available. A dorsal approach was found to be the safest and most reproducible technique in a cadaveric model. These findings may be applied in the clinical setting, where steroid injections are used for the diagnosis and treatment of STT arthritis.
